# Association of polymorphic variants in serotonin re-uptake transporter gene with Crohn's disease: a retrospective case-control study

**DOI:** 10.3325/cmj.2018.59.232

**Published:** 2018-10

**Authors:** Katja Grubelic Ravić, Frane Paić, Boris Vucelić, Marko Brinar, Silvija Čuković-Čavka, Nada Božina, Željko Krznarić, Mirjana Kalauz, Dino Bešić, Tamara Nikuševa Martić

**Affiliations:** 1 Department of Gastroenterology, University Hospital Center Zagreb, Zagreb, Croatia; 2 Laboratory for Epigenetic and Molecular Medicine, Department of Biology and Medical Genetics, Zagreb University School of Medicine, Zagreb, Croatia; 3 Clinical Institute of Laboratory Diagnoses, University Hospital Center Zagreb, Zagreb, Croatia

## Abstract

**Aim:**

To analyze the distribution of *SLC6A4* gene polymorphisms in Crohn’s disease (CD) patients and their association with the disease.

**Methods:**

We evaluated the presence/absence of promoter (*5-HTTLPR, rs25531*) and intron 2 (*STin2 VNTR*) polymorphic variants of *SLC6A4* gene in a retrospective case-control study including 192 CD patients and 157 healthy controls (HC). Genotyping was performed by polymerase chain reaction. The association of polymorphisms with CD and its clinical subtypes was analyzed using χ^2^ and Fisher exact test, binary logistic regression, and haplotype analysis.

**Results:**

CD patients and healthy controls had similar sex (88 [45.8%] vs 84 [53.5%] women, respectively; *P* = 0.154) and age (41.3 ± 12.8 years vs 41.7 ± 8.8 years, respectively, *P* = 0.091) distribution. Significant differences were observed in the *STin2* genotype and allele distribution between CD patients and healthy controls (*P* = 0.003 and *P* = 0.002, respectively) and between the corresponding female subgroups (*P* = 0.004 and *P* = 0.007, respectively), with a significant negative association of biallelic ss (*STin2.9* and *Stin2.10*) *STin2* genotype with CD (*P* = 0.013, age- and sex-adjusted odds ratio [OR] 0.5, 95% confidence interval [CI] 0.29-0.86; women: *P* = 0.006, age-adjusted OR 0.32, 95% CI 0.14-0.72) and a significantly higher *S-STin2.12* (*5-HTTLPR/rs25531*: *S-STin2: STin2.12*) haplotype distribution in CD patients (*P* = 0.004, OR 1.62, 95% CI 1.16-2.26). There was no significant association between *5-HTTLRP* and *rs25531* genotype or allele frequencies and CD and between any *SLC6A4* polymorphic loci with clinical CD subtypes.

**Conclusion:**

*STin2 VNTR* polymorphism of *SLC6A4* gene may contribute to CD pathogenesis.

Inflammatory bowel disease (IBD), with its constituent clinical phenotypes, Crohn’s disease (CD) and ulcerative colitis (UC), represents a major relapsing gastrointestinal (GI) disorder, with a combined incidence of 2-20 per 100 000 individuals in the developed countries (1-4). IBD susceptibility is influenced by a variety of factors, including genetic polymorphism, GI motility, stress response, visceral hypersensitivity, abnormal immune response, and its reaction to enteromicrobial pathogens (1-4).

Neuroimmunological interactions are also important because various pro- and anti-inflammatory cytokines may affect neuronal activity and the release of neurotransmitters influencing the activity of immuno-effector cells in the GI tract. The best characterized among them is serotonin (5-HT), 90% of which is produced and secreted by intestinal enterochromaffin cells (5-8). Alterations in 5-HT biosynthesis, quantity, release, or clearance are important for both sensory signal transduction in GI motility and the development of visceral hypersensitivity (5-8). 5-HT is also a chemotactic molecule and may promote lymphocyte activation and secretion of pro-inflammatory cytokines (5-8). Therefore, upon its release and binding to targeted receptors, 5-HT action must be rapidly terminated. This is maintained by the action of serotonin re-uptake transporter (SERT), expressed by serotonergic neurons and the mucosal enterocytes (5-8).

Animal models and studies on human cell lines and tissue indicate increased 5-HT availability and reduced SERT expression in the inflamed colon, accompanied with an increased expression of inflammatory genes, thus supporting the idea that the loss of SERT gene (*SLC6A4*) transcription can either cause intestinal inflammation or result from it (8,9). *SLC6A4* gene polymorphisms have also been linked with its translation and expression (10,11).

The most extensively studied among them are *5-HTTLPR, rs25531 (A/G)* single nucleotide (SNP), and *STin2 VNTR* (variable number of tandem repeats) polymorphic regions found in the promoter (*5-HTTLPR and rs25531*) and intron 2 region (*STin2 VNTR*) of *SLC6A4* gene (Figure 1) (12-19).

**Figure 1:**
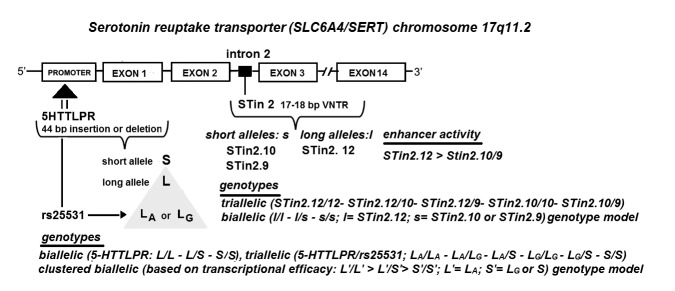
Location of SLC6A4 gene polymorphic regions and their genotype combinations examined in this study. *SLC6A4* gene is affected by the insertion/deletion variation of the promoter region *5-HTTLPR,* resulting in two alleles, long *L* and small *S*. This promoter region also functionally couples with the common *rs25531 (A/G)* single nucleotide polymorphism (SNP), resulting in alleles *L_A_* and *L_G_*. *L_A_* allele is associated with the higher transcriptional activity, whereas the *L_G_* allele exhibits lower serotonin uptake and the transcriptional activity equivalent to *S* allele of the *5-HTTLPR* polymorphic region. Transcriptional activity of *SLC6A4* gene is further modulated by the enhancer activity of *STin2* polymorphic region, 17–18 bp long variable number of tandem repeats (VNTR), found in intron 2. The *STin2* allelic variants were identified as 10-repeat and 12-repeat alleles that have been identified in all ethnicities, and the less common 9-repeat allele found only in individuals of European or African descent. Thus, promoter polymorphic region may be presented in biallelic, triallelic, and clustered biallelic genotype model while *STin2 VNTR* region may be presented in triallelic and biallelic genotype forms. Rare allele forms (eg, *XL* or *STin2.7*) not encountered in this study, are not presented.

Sikander et al (20) has reported a significant association of *5-HTTLPR* polymorphism with UC and microscopic colitis (MC). However, the association between *5-HTTLPR* and CD has never been determined. The same was true for other *SLC6A4* polymorphic regions in any form of IBD (11,20). Therefore, the aim of this study was to compare genotype and allele frequencies of *5-HTTLPR, rs25531*and *STin2 VNTR* polymorphic regions in CD patients and healthy control participants and investigate their association with CD. Given the crucial role of SERT protein in the regulation of intestinal 5-HT availability, we hypothesized that the *SLC6A4* polymorphism may contribute to the genetic predisposition for the development of CD.

## PATIENTS AND METHODS

### Patients

This retrospective case-control study included all 192 consecutive CD patients diagnosed between December 2012 and 2016 at the University Hospital Center Zagreb (Table 1). CD diagnosis was established according to standard endoscopic, radiological, and histopathological criteria (21). The healthy control group consisted of 157 participants with no family history of IBD or irritable bowel disease. They were selected from 217 completely healthy voluntary participants (hospital employees or their acquaintances) and age- and sex-matched to CD patient group. Genetically related voluntary control participants and participants who were incompatible according to age (outside the age range of CD group members) were excluded from further analysis. All participants signed the informed consent for study participation and data publication. The study protocol was approved by the Ethics Committee of the University Hospital Center Zagreb (Approval number 04/31-JG; contract number 108-1081874-1917, start date January 1, 2007).

**Table 1:** Clinical characteristics of patients with Crohn’s disease (CD) analyzed in the study

	No. (%) of CD patients		
Characteristics	total	women	men
Age at diagnosis (years)			
≤16	21 (10.93)	5 (23.8)	16 (76.2)
17-41	153 (79.69)	76 (49.7)	77 (50.3)
>41	18 (9.38)	7 (38.9)	11 (61.1)
Disease location			
ileum	60 (31.3)	29 (48.3)	31 (51.7)
colon	34 (17.7)	14 (41.2)	20 (58.8)
ileocolon	91 (47.4)	41 (45.1)	50 (54.9)
upper gastrointestinal (UGI) tract	29 (15.1)	17 (58.6)	12 (41.4)
no UGI location	157 (81.8)	68 (43.3)	89 (56.7)
Disease type			
inflammatory	89 (46.4)	40 (44.9)	49 (55.1)
stricturing	64 (33.3)	31(48.4)	33 (51.6)
penetrating	39 (20.3)	17(43.6)	22 (56.4)
Perianal disease			
yes	63 (32.8)	24 (38.1)	39 (61.9)
no	129 (67.2)	64(49.6)	65 (50.4)
Extra-intestinal manifestation			
yes	43 (22.4)	23 (53.5)	20 (44.7)
no	134 (69.8)	57 (42.54)	77 (57.46)
Family history			
yes	17 (8.85)	8 (47.1)	9 (52.9)
no	175 (91.15)	80 (45.71)	95 (54.29)
Surgery			
yes	105 (54.69)	45 (42.9)	60 (57.1)
no	87 (45.31)	43 (49.4)	44 (50.6)

### DNA extraction and genotyping

Blood samples were collected by venipuncture in EDTA BD Vacutainer® blood collection tubes (Becton, Dickinson and Company, Franklin Lakes, NJ, USA), and genomic DNA was isolated from peripheral blood leukocytes using the salting out procedure (22). Study participants were genotyped for both promoter and intron 2 polymorphisms of the *SLC6A4* as previously described (22-24). Biallelic *5-HTTLRP* variants were discriminated with some modifications as previously described (22-24). The following primers were used: forward 5′-ATG CCA GCA CCT AAC CCC TAA TGT-3′, reverse 5′-GGA CCG CAA GGT GGG CGG GA-3′. The *5-HTTLPR* polymorphic region was subdivided into functional variants (*S, LA, LG*) by *rs25531 (A/G) SNP* using a TaqMan qPCR procedure according to Hu et al (25). Amplification was performed in a 7500 qPCR System under the following conditions: at 50°C for 2 minutes, at 95°C for 15 seconds, and at 62.5°C for 1.5 minutes. Genotype sequencing/capillary electrophoresis (ABI PRISM 3100 Genetic Analyzer, TermoFisher Scientific, Waltham, MA, USA) was performed to acquire controls. The *STin2 VNTR* polymorphism was discriminated using the method described by Ito et al (26). For the PCR reaction, the following primer pair was used: forward 5′-GTCAGTATCACAGGCTGCGAG-3′, reverse 5′-TGTTCCTAGTCTTACGCCAGTG-3′.

### Statistical analysis

The normality of data distribution was analyzed by Shapiro-Wilk test. Data are presented as frequencies or means with standard deviations (±SD). The groups were compared using *t* test or Mann-Whitney test for parametric and non-parametric continuous variables, respectively, and Pearson χ^2^ test for categorical variables. Conformity of genotype distributions to Hardy–Weinberg equilibrium was assessed using goodness-of-fit χ^2^ test. The difference in allele frequency was determined by using either χ^2^ or Fisher exact test as appropriate. Crude and adjusted odds ratios (OR) (controlling for age and sex) and the corresponding 95% confidence intervals (CI) determined by binary logistic regression were also used to test the association between CD and genotype variants of both promoter and intron 2 *SLC6A4* gene polymorphisms. Association analysis based on patient sex, or clinical CD subtypes such as age at diagnosis, localization, and disease behavior were also performed. Considering that *LG* allele has the same transcriptional activity as the *S* allele, the triallelic *5-HTTLPR-rs25531* genotypes were also coded in a clustered biallelic form (*L'L', L'S'* and *S'S'*; Figure 1). Likewise, the triallelic *STin2 VNTR* polymorphism was also coded in a biallelic (*ll - ls - ss;* Figure 1) genotype form. All biallelic genotype forms were analyzed under the codominant, dominant, and recessive model. Construction of haplotypes, linkage disequilibrium (LD), and haplotype association analysis were calculated by permutation test using the online SHEsisPlus software (27).

The power analysis (to compute *a priori* sample size and the *post-hoc* power of the study) was performed using G*Power software (version 3.1.9.2 for Windows) (28). For the analysis of *5-HTTLRP* genotype frequencies with a χ^2^ goodness-of-fit test with the small effective size (w = 0.2, df = 2); power (1-β) = 0.80, and α = 0.017 (Bonferroni corrected *P* value), the required total sample size was 313 and actual sample size was 349. *Post-hoc* computed achieved power (1-β) of analysis was 0.085 (w = 0.2), 0.997 (w = 0.3; medium effective size), 1.00 (w = 0.5; large effective size) for genotype distribution, and 0.80 for allele frequency analysis (w = 0.2 - 0.5; df = 1). For the *a priori* analysis of *STin2 VNTR* genotype frequencies with a χ^2^ goodness-of-fit test with the small effective size (w = 0.23, df = 4); power (1-β) = 0.80 and α = 0.008 (Bonferroni corrected *P* value), the required total sample size was 329, and the *post-hoc* achieved power (1-β) of analysis was 0.83-1.00 for genotype distribution and 0.99-1.00 (w = 0.2 - 0.5; df = 2) for allele frequency analysis. Furthermore, with a power of 0.80 we were able to detect an effect size of 0.223 for a significant difference in *STin2 VNTR* genotype frequencies. The two-tailed *P* < 0.05 was considered significant and corrected according to Bonferroni procedure (the corrected level of significance is: *P*c = 0.05/N; N - number of independent tests). All reported *P* values were uncorrected unless stated otherwise. Statistical analysis was performed using SPSS Statistics software trial version (IBM Corp., Armonk, NY, USA), unless stated otherwise.

## RESULTS

### Population characteristics

There were 88 (45.8%) women and 104 (54.2%) men in the CD group, and 84 (53.5%) women and 73 (46.5%) men in the control group (Pearson χ2 = 2.033, *P* = 0.154). The groups were also comparable according to age (mean±SD: 41.3 ± 12.8 years vs 41.7 ± 8.7 years, respectively; Mann-Whitney test, *P* = 0.091).

### Association analysis

According to the Hardy-Weinberg equilibrium, there was no deviation in the 5-HTTLPR, STin2, and rs25531 genotype distribution in CD group (5*-HTTLPR*: χ2 = 0.557, df = 1, *P* = 0.756; *rs25531*: χ2 = 1.151, df = 3, *P* = 0.949; *STin2*: χ2 = 0.585, df = 3, *P* = 0.964) and healthy control group (*5-HTTLPR*: χ2 = 0.156, df = 1, *P* = 0.924; *rs25531*: χ2 = 2.358, df = 3, *P* = 0.797; *STin2 VNTR*: χ2 = 7.224, df = 3, *P* = 0.124). Furthermore, allele and genotype frequencies of all polymorphic loci in the control group corresponded well to the previously published data for Croatian and other European populations (18,29-31).

CD group and control group showed a different distribution of *STin2 VNTR* allele frequencies, which remained significant after Bonferroni correction (Table 2). This was also the case for women with CD (χ2 = 9.850, df = 2, *P* = 0.007) and their healthy female controls (data not shown). In addition, *STin2.12* allele showed a positive and *STin2.9* allele a negative association with CD both in the overall (Table 2) patient sample and in the subgroup of female patients (data not shown). However, the association remained significant only for the rare *STin2.9* allele in the overall study sample and the subgroup of female patients after Bonferroni correction (Table 2). No significant association following Bonferroni correction was found between *STin2 VNTR* allele frequency and clinical CD subtypes (data not shown).

**Table 2:** *5-HTTLPR, STin2 VNTR* and *rs25531* SNP allele frequency distribution in patients with Crohn’s disease and healthy controls*

Polymorphism	No. (%) of participants		Pearson χ^2^		Individual comparisons		
	CD	controls	χ^2^	*P*^‡^	χ2	*P^‡^*	OR (95% CI)^§^
*5-HTTLRP*							
*L*	231 (60.2)	208 (66.2)	2.74	0.098	2.74	0.098	0.77 (0.56-1.05)
*S*	153 (39.8)	106 (33.8)			2.74	0.098	1.30 (0.95-1.77)
*rs25531*							
*L_A_*	212 (55.2)	185 (58.9)	3.76	0.152	0.97	0.325	0.86 (0.64-1.16)
*L_G_*	19 (5.0)	23 (7.3)			1.73	0.188	0.66 (0.35-1.23)
*S*	153 (39.8)	106 (33.8)			2.74	0.097	1.30 (0.95-1.77)
*STin2 VNTR*							
*12*	242 (63.0)	174 (55.4)	12.03	0.002†	4.15	0.042	1.37 (1.01-1.86)
*10*	141 (36.7)	130 (41.4)			1.59	0.207	0.82 (0.61-1.12)
*9*	1 (0.3)	10 (3.2)				0.002	0.08 (0.01-0.62)

*CD – Crohn’s disease; *5-HTTLPR* – serotonin reuptake transporter (*SLC6A4*) length polymorphic region; *STin2 VNTR* – variable number of tandem repeats (VNTR) found in intron 2 of *SLC6A4* gene; *rs25531* – single nucleotide (SNP) polymorphism found in the background of longer *5-HTTLPR* allele variant; *L* – long *5-HTTLPR* allele; *S* – short *5-HTTLPR* allele; *L_A_* – long *5-HTTLPR* allele associated with the higher transcriptional activity; *L_G_* – long *5-HTTLPR* allele exhibiting lower serotonin uptake and transcriptional activity equivalent to the short (*S*) allele of the *5-HTTLPR* polymorphic region; *12, 10, 9* – *STin2 VNTR* alleles; OR – odds ratio; CI – confidence interval.

†Bonferroni correction *P_c_* = 0.05/3 = 0.017.

**‡**Bonferroni non-adjusted *P* values.

§Logistic regression adjusted for age and sex.

There was no significant association in *5-HTTLRP* and *rs25531* allele frequency between the CD and control group or between the corresponding sex subgroups (data not shown). The results remained the same after the analysis for clinical CD subtypes (data not shown). Nevertheless, we did observe a positive, although non-significant, association of S allele of 5*-HTTLRP* and *rs25531* polymorphism with CD (Table 2).

Pearson χ^2^ test showed significant differences in *STin2* genotype distribution (Table 3) between CD patients and healthy controls (*P* = 0.003) and between the corresponding female (χ2 = 15.326, df = 4, *P* = 0.004) subgroups (data not shown). This was even more pronounced when carriers of the rare *STin2* genotype forms (*STin2 12/9 + STin2 10/9*; Fisher exact test for CD vs controls; *P* = 0.002) were grouped together (overall CD: χ2 = 15.720, df = 3, *P* = 0.001; female subgroup: χ2 = 15.326, df = 3, *P* = 0.002). Furthermore, *STin2 12/12* and *Stin2 12/10* genotypes were more common, while *STin2 10/10* genotype was less common among CD patients and controls. The same was true when we analyzed the corresponding female subgroups only (data not shown). However, even at very liberal correction for multiple comparisons of *Pc* = 0.008 (*Pc* = 0.05/N – number of genotypes analyzed), we did not find any significant difference in the distribution of individual *STin2 VNTR* genotypes between CD and controls (Table 3).

**Table 3:** Logistic regression analysis of *STIN2 VNTR* genotype distribution in patients with Crohn’s disease and healthy controls*

Polymorphism	No. (%) of participants		Logistic regression			
	CD	controls	crude OR (95% CI)	*P^†^*	OR (95% CI)^‡^	*P^†^*
***STin2 VNTR* triallelic**^§^				0.038^II^		0.043^II^
codominant model A						
*12/12*	76 (39.6)	55 (35.0)	reference 1.0		reference 1.0	
*12‎/10*	89 (46.4)	57 (36.3)	1.13 (0.7-1.83)	0.618	1.15 (0.71-1.86)	0.570
*10‎/10*	26 (13.5)	35 (22.3)	0.54 (0.29-0.99)	0.048	0.55 (0.30-1.02)	0.056
*12‎/9*	1 (0.5)	7 (4.5)	0.10 (0.01-0.86)	0.036	0.11 (0.013-0.89)	0.040
*10‎/9*	0 (0.0)	3 (1.9)	NA	0.990	NA	0.100
codominant model B^¶^				0.008**		0.009**
*12/12*	76 (39.6)	55 (35.0)	reference 1.0		reference 1.0	
*12‎/10*	89 (46.4)	57 (36.3)	1.13 (0.7-1.83)	0.618	1.15 (0.71-1.86)	0.600
*10‎/10*	26 (13.5)	35 (22.3)	0.54 (0.29-0.99)	0.048	0.55 (0.3-1.02)	0.060
*9/*others	1 (0.5)	10 (6.4)	0.07 (0.01-0.58)	0.014**	0.073 (0.01-0.59)	0.014**
***STin2 VNTR* biallelic**				0.041^II^		0.045^II^
codominant model						
*l/l*	76 (39.6)	55 (35.0)	2.02 (1.10-3.71)	0.023	1.98 (1.08-3.64)	0.028
*l/s*	90 (46.9)	64 (40.8)	2.06 (1.14-3.72)	0.017	2.05 (1.13-3.71)	0.018
*s/s*	26 (13.5)	38 (24.2)	reference 1.0		reference 1.0	
dominant						
*l/l*	76 (39.6)	55 (35.0)	reference 1.0		reference 1.0	
*l/s-s/s*	116 (60.4)	102 (65.0)	0.82 (0.53-1.27)	0.380	0.84 (0.54-1.30)	0.400
recessive						
*l/l-l/s*	166 (86.5)	119 (75.8)	reference 1.0		reference 1.0	
*s/s*	26 (13.5)	38 (24.2)	0.49 (0.28-0.85)	0.011**	0.5 (0.29-0.86)	0.013**

*CD – Crohn’s disease; *STin2 VNTR* – variable number of tandem repeats (VNTR) found in intron 2 of serotonin reuptake transporter (*SLC6A4*) gene; OR – odds ratio; CI – confidence interval. *l* – Long (12) *STin2* alleles; *s* – short (10, 9) *STin2* alleles; *12, 10, 9*: *STin2 VNTR* alleles.

†Bonferroni non-adjusted *P* values.

‡Logistic regression adjusted for age and sex.

§Overall *STin2 VNTR* genotype distribution in CD patients and healthy controls: χ2 = 15.857, df = 4, *P* = 0.003.

IIGlobal *P* value for logistic regression analysis.

¶Rare genotype variants grouped together.

**Significant *P* values (<*P_c_*). Bonferroni correction for genotype distribution analysis *P_c_* = 0.008 (0.05/6 – number of genotypes) and for logistic regression analysis *P_c_* = 0.017 (0.05/3 – number of genetic model comparisons).

In logistic regression analysis, the co-dominant triallelic *STin2* genotype model did not show a significant association of individual *STin2* genotypes with CD (Table 3). However, when men and women were analyzed together under the recessive biallelic model (Table 3) or when women only were analyzed (data not shown), carriers of biallelic *ss* (*s = STin2 10* or *STin2 9*), *STin2* genotype form exhibited a significant negative association with CD compared with *ll* and *ls* carriers (female patients: Wald = 7.564, df = 1, *P* = 0.006, age-adjusted OR = 0.32, 95% CI = 0.14-0.72), which remained significant after sex and/or age adjustment (female patients: Wald = 7.564, df = 1, *P* = 0.006, OR adjusted by age = 0.32, 95% CI = 0.14-0.72). Nevertheless, only the variation of co-dominant *STin2* model in which the rare genotypes (*STin2 12/9* and *10/9*) were grouped together showed significant global *P* values in crude and adjusted logistic regression analysis, and only when male and female patients were analyzed together (Table 3).

No significant difference was observed in *5-HTTLRP* and *rs25531* genotype distribution between CD group and controls (Table 4) or between sex subgroups and clinical CD subtypes (data not shown). The same was found when biallelic *5-HTTLRP* and *rs25531* genotype forms were analyzed under the codominant, dominant, or recessive genetic model. Nevertheless, we did observe more frequent, although non-significant, distribution of heterozygous (*LS, LALG, L'S'*) and homozygous *SS* genotype forms in the CD group (Table 4).

**Table 4:** Logistic regression analysis of *5-HTTLRP* and *rs25531* genotype distribution in patients with Crohn’s disease and healthy controls*

Polymorphism	No. (%) of participants		Logistic regression			
	CD	controls	crude OR (95% CI)	*P^†^*	OR (95% CI) ^‡^	*P^†^*
***5-HTTLRP biallelic***^§^				0.180^II^		0.200^II^
codominant						
*L/L*	67 (34.9)	70 (44.6)	reference 1.0		reference 1.0	
*L/S*	97 (50.5)	68 (43.3)	1.50 (0.94-2.35)	0.090	1.48 (0.94-2.35)	0.090
*S/S*	28 (14.6)	19 (12.1)	1.54 (0.79-3.02)	0.210	1.52 (0.77-2.98)	0.230
dominant						
*L/L*	67 (34.9)	70 (44.6)	reference 1.0		reference 1.0	
*L/S-S/S*	125 (65.1)	87 (55.4)	1.50 (0.97-2.31)	0.070	1.49 (0.97-2.30)	0.070
recessive						
*L/L-L/S*	164 (85.4)	138 (87.9)	reference 1.0		reference 1.0	
*S/S*	28 (14.6)	19 (12.1)	1.24 (0.66-2.32)	0.500	1.22 (0.65-2.29)	0.530
***5-HTTLRP/rs25531 triallelic***^¶^				0.670^II^		0.670^II^
codominant						
*L_A_/L_A_*	57 (29.7)	57 (36.3)	reference 1.0		reference 1.0	
*L_A_/L_G_*	10 (5.2)	11 (7.0)	0.91 (0.36-2.31)	0.840	0.91 (0.36-2.31)	0.840
*L_A_/S*	88 (45.8)	60 (38.2)	1.47 (0.90-2.40)	0.130	1.47 (0.90-2.41)	0.130
*L_G/_S*	9 (4.7)	8 (5.1)	1.13 (0.41-3.12)	0.820	1.07 (0.38-2.98)	0.900
*L_G_/L_G_*	0 (0.0)	2 (1.3)	NA	0.990	NA	0.100
*S/S*	28 (14.6)	19 (12.1)	1.47 (0.74-2.93)	0.270	1.46 (0.73-2.90)	0.290
***5-HTTLRP/rs25531 biallelic***				0.410^II^		0.410^II^
codominant						
*L'/L'*	57 (29.7)	57 (36.3)	reference 1.0		reference 1.0	
*L'/S'*	98 (51.0	71 (45.2)	1.38 (0.86-2.23)	0.190	1.39 (0.86-2.24)	0.180
*S'/S'*	37 (19.3)	29 (18.5)	1.28 (0.69-2.35)	0.430	1.25 (0.68-2.31)	0.470
dominant						
*L'/L'*	57 (29.7)	57 (36.3)	reference 1.0		reference 1.0	
*L'/S'- S'/S'*	135 (70.3)	100 (63.7)	1.35 (0.86-2.12)	0.190	1.346 (0.86-2.11)	0.200
recessive						
*L'/L'- L'/S'*	155 (80.7)	128 (81.5)	reference 1.0		reference 1.0	
*S'/S'*	37 (19.3)	29 (18.5)	1.05 (0.61-1.81)	0.850	1.032 (0.60-1.77)	0.910

*CD – Crohn’s disease; *5-HTTLPR* – serotonin reuptake transporter (*SLC6A4*) length polymorphic region; *rs25531* – single nucleotide (SNP) polymorphism found in the background of longer *5-HTTLPR* allele variant; *L^’^: L_A;_ S’: L_G_* or *S* (clustered biallelic *5-HTTLRP/rs25531* model based on transcriptional activity: *L’/L’>L’/S’>S’/ S’*) OR – odds ratio, CI – confidence interval.

†Bonferroni non-adjusted *P* values; Bonferroni correction for genotype distribution analysis *P_c_* = 0.0083 (0.05/6 - number of genotypes) and for logistic regression analysis *P_c_* = 0.0166 (0.05/3 - number of genetic model comparisons).

‡Logistic regression adjusted for age and sex.

§Overall *5-HTTLRP* genotype distribution between CD and healthy control group: χ2 = 3.410, df = 2, *P* = 0.182;

IIglobal *P* value of logistic regression analysis.

¶Overall triallelic *5-HTTLRP/rs25531* genotype distribution between CD and healthy control group: χ2 = 5.674, df = 5, *P* = 0.339.

### Haplotype analysis

**Figure 2:**
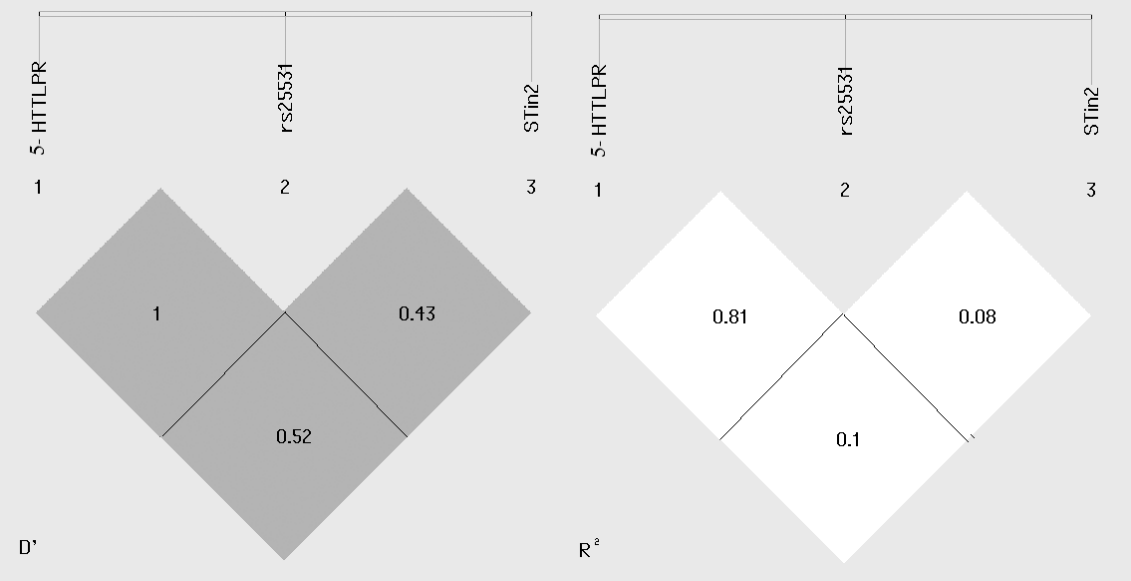
Linkage disequilibrium (LD) in SLC6A4 gene with D' and r2 values.

*5-HTTLPR* and *rs25531* polymorphic region were in strong linkage disequilibrium, while LDs between them and *STin2 VNTR* polymorphic region were relatively weak (Figure 2). After Bonferroni correction, only *S-S-STin2.12* haplotype showed a significantly higher frequency in CD group (Table 5). The higher frequency in CD group was also revealed for *L-LG-STin2.10* haplotype, but it was not significant (Table 5).

**Table 5:** The frequency of *SLC6A4* haplotypes in patients with Crohn’s disease and their healthy controls*

*SLC6A4* haplotypes			No. (%) of participants		χ2	*P^†^*	OR (95% CI)^†^
*5-HTTLRP*	*rs25531*	*STin2 VNTR*	CD	controls			
*L*	*L_A_*	*STin2 10*	110 (28.6)	97 (30.8)	0.417	0.518	0.89 (0.65-1.24)
*S*	*-*	*STin2 12*	131 (34.1)	76 (24.2)	8.133	0.004^‡^	1.62 (1.16-2.26)
*L*	*L_A_*	*STin2 12*	102 (26.5)	88 (28.0)	0.186	0.665	0.93(0.66-1.30)
*L*	*L_G_*	*STin2 10*	9 (2.3)	6 (1.9)	0.153	0.694	1.23 (0.43-3.50)
*S*	*-*	*STin2 10*	22 (5.7)	27 (8.5)	2.179	0.139	0.65 (0.36-1.16)
*L*	*L_G_*	*STin2 12*	9 (2.3)	10 (3.1)	0.461	0.496	0.73 (0.29-1.82)
*L*	*L_G_*	*STin2 9*	1 (0.2)	7 (2.2)	5.910	0.015	0.11 (0.01-0.94)
*S*	*-*	*STin2 9*	0 (0.0)	3 (0.9)	3.684	0.054	NA

* CD – Crohn’s disease; *5-HTTLPR* – serotonin reuptake transporter (SERT/*SLC6A4*) length polymorphic region; *STin2 VNTR* – variable number of tandem repeats (VNTR) found in intron 2 of *SLC6A4* gene; *rs25531* – single nucleotide (SNP) polymorphism found in the background of longer *5-HTTLPR* allele variant; *L_G_* – Long *5-HTTLPR* allele exhibiting lower serotonin uptake and transcriptional activity equivalent to the short (*S*) allele of the *5-HTTLPR* polymorphic region; *L_G_* – Long *5-HTTLPR* allele exhibiting lower serotonin uptake and transcriptional activity equivalent to the short (*S*) allele of the *5-HTTLPR* polymorphic region; Global result – Pearson χ^2^ = 18.29, Pearson *P* = 0.011; OR – odds ratio; CI – confidence interval.

†Bonferroni non-adjusted *P* values and confidence intervals; Bonferroni correction for haplotype analysis: *P_c_* = 0.006 (0.05/8 – number of haplotype comparisons).

‡Significant *P* value (<*P_c_*).

## DISCUSSION

Our data revealed a significant difference in *STin2 VNTR* genotype and allele distribution between the overall CD group and healthy controls and between the female patient group and the corresponding control group. There was also a significant negative association of biallelic ss (*STin2. 10/10* and *STin2. 10/9* vs *STin2. 12/12, 12/10* and *12/9* combined) genotype form and CD. We also found a significantly higher *S-STin2.12* (*5-HTTLPR/rs25531*: *S - STin2: STin2.12*) haplotype distribution among CD patients. However, we did not find any association between *5-HTTLRP* and *rs25531* genotype or allele frequencies and CD or between any *SLC6A4* polymorphic loci and clinical subtypes of CD. Nevertheless, our results indicate that *STin2 VNTR* polymorphism of *SLC6A4* gene may contribute to CD pathogenesis.

Altered function and down-regulation of SERT protein expression paralleled with abnormal 5-HT concentration, both locally in epithelial layer and in the circulation, has been documented in several human GI inflammatory conditions including CD, UC, and MC, as well as diverticulitis and active celiac disease (32-34). This was also documented in several animal models of intestinal inflammation (35-39). Pro-inflammatory mediators and growth factors released during IBD may down-regulate *SLC6A4* transcription and decrease SERT protein expression and function (40-43).

Another possibility is that some individuals with IBD have a genetic predisposition leading to altered SERT expression with consequent changes in gastrointestinal 5-HT levels, which contributes indirectly to pro-inflammatory conditions in the affected intestinal mucosa.

However, despite the recognition that intestinal inflammatory diseases, such as CD, can have a strong genetic component, polymorphisms so far linked with SERT transporter have not been associated with CD.

Furthermore, in previous reports on GI diseases only *STin2 VNTR* was observed as an attractive candidate for a possible association of the SERT with IBS (10,44). Although IBD and IBS are usually viewed as dichotomous conditions, they exhibit similar alterations in serotonergic-signaling mechanisms (33,44-46). Due to the development of IBS symptoms in IBD patients in remission and the clinical overlap between IBD and IBS, some authors even argued that they may represent clinical manifestations of a pathophysiologic spectrum of the same disease (47,48). Several population studies showed that IBS patients have an increased risk of becoming IBD patients than patients without prior IBS history, and this effect may be greater for CD (49,50).

Reports regarding the link between *STin2* polymorphism and IBS were controversial and inconclusive, with most of them indicating no association with IBS or its clinical subtypes (47). However, Wang et al (51) found that IBS patients had a greater frequency of *STin2.12/10* and a lower frequency of *STin2.12/12* genotype compared to controls, but identified no significant difference in STin2 polymorphism among different clinical subtypes of IBS.

Current research in functional implications of the *STin2* polymorphism is also inconclusive. It is known that *STin2* acts as a transcriptional regulator and has allele-dependent enhancer-like properties that may influence tissue-specific regulation of *SLC6A4* gene (18,52). However, it seems that individual *SLC6A4* polymorphisms have a weak influence compared with the combined effect of *5-HTTLPR* and *STin2* region (14,18,52,53). Therefore, their allelic combinations should be identified before concluding about functional and phenotypic associations. LD between these two loci, ranging from moderate (European) to very strong (Native Americans), was found in most of the studied populations (31). In addition, a partial linkage of *STin2.12* allele with S allele of *5-HTTLPR* (*S12* haplotype combination) has been reported and had stronger enhancer-like properties on *SLC6A4* transcription than *L10* or *S10* haplotype (53,54). We found a significantly higher distribution of *S12* haplotype in CD group, showing its positive association with disease occurrence. However, since we did not measure the level of SERT expression or 5-HTT plasma or tissue levels, we were not able to indicate any functional consequences of this finding.

We did not confirm the association of *5-HTTLRP* and *rs25531* with CD occurrence, but we did observe the tendency toward higher frequencies of heterozygote *5-HTTLRP* and *rs25531* genotype forms in CD group. This effect of molecular heterosis was also described in some other studies of *5-HTTLPR* (55-57).

To the best of our knowledge, there are no reports analyzing the relationship between the promoter polymorphic regions of the *SLC6A4* gene with CD. Sikander et al (20) demonstrated a potential association between *5-HTTLPR* polymorphism and UC and MC by finding a significantly lower frequency of *SS* vs non-*SS* (*LL* and *LS* combined) *5-HTTLPR* genotypes in MC patients or UC patients in remission and significantly higher serum 5-HT levels (*SS>LS>LL*) in these two patient groups. This was especially the case in MC *LS* and *SS* genotype carriers compared with controls (20). In addition, UC patients with active disease and SS genotype also had increased 5-HT levels compared with control subjects expressing the same genotype (20). Similar to our results in CD patients, they did not observe any significant differences in *5-HTTLPR* genotype and allele distributions between UC patients with active disease and healthy controls or between male and female patient subgroups (20). Likewise, Shiotani et al (34) also observed no significant association of *5-HTTLPR* variants and UC. Our results were contrary to the findings of Sikander et al (20), as we found a higher frequency of *SS* genotype in CD group.

The observed discrepancies are possibly due to heterogeneous background of CD and other forms of IBD and/or ethnical or regional differences between the studied groups. They are also consistent with the previously mentioned hypothesis that *5-HTTLPR* might represent only one of the contributing polymorphic factors responsible for the disease occurrence.

Regarding the IBS and *5-HTTLRP* polymorphism, different associations were reported, but the results were contradictory and inconclusive even for the same population under the study. The most recent meta-analysis found no significant association between *5-HTTLPR* and IBS in overall population, while in the IBS subtype- and ethnic subgroup-based analysis the *LL* genotype was demonstrated as a risk factor for constipation predominant IBS (58).

With respect to rs25531 polymorphism, we found only two studies examining its association with IBS, and only one reported a positive association with disease occurrence with three times higher odds ratio for the LG allele distribution in IBS patients compared to controls (59,60).

There are several limitations to our study. The sample size was too small for a comprehensive genotype analysis, so the results cannot be generalized and should be interpreted cautiously. Second, we did not determine the 5-HT levels and were unable to evaluate the functional consequences of individual genotype or haplotype variants on SERT expression. Third, association studies of unrelated individuals, such as ours, warrant cautious interpretation as unknown sources of population stratification may affect the results.

In conclusion, we demonstrated significant differences in *STin2* genotype and allele distribution between CD patients and healthy controls, and a negative association of biallelic ss *STin2* genotype variant and a positive association of *S12* haplotype with CD disease. Further large-scale studies in this and other populations aimed to confirm the obtained results and decipher the exact functional role of *STin2 VNTR* polymorphic region in intestinal inflammatory diseases are warranted.
